# Long-term outcomes of acute coronary syndrome in young adults: findings from GULF RACE-2

**DOI:** 10.1186/1753-6561-6-S4-P23

**Published:** 2012-07-09

**Authors:** M Almohammadi, A Hersi, KF Alhabib, AA Alsheikh-Ali, K Sulaiman, H Alfaleh, S Alsaif, W Almahmeed, N Asaad, H Amin, A Al-Motarreb, J Al Suwaidi

**Affiliations:** 1 King Saud University, Saudi Arabia

## Introduction

Long term outcome of young patients presenting with acute coronary syndrome (ACS) has not been described in the Middle East. Accordingly we sought to evaluate the risk factors, presentations, clinical assessments and long-term outcome of these patients.

## Methods

We used a multi-center, prospective Gulf RACE-2 (Registry of Acute Coronary Events) to determine the long term outcome of young patients with ACS.

## Results

The study enrolled 7930 ACS patients from 65 hospitals in 6 Arabian Gulf countries, during the period of October 2008 to June 2009. Of these, 686 (8.7%) were 40 years of age or younger, with mean age of 36 +/- 4 years. Compared to older patients, the young patients had higher prevalence of STEMI (62% vs 42.9%, P<0.001) and male gender (91.3% vs 77.6, P<0.001). The major risk factor was smoking (61.5% vs 33.2%, P<0.001). Furthermore, the percentage of diabetes, hypertension and hyperlipidemia were significantly low in younger age group (18% vs 42.2%), (21.4% vs 50.2%) and (22% vs 38.9%), respectively. P<0.001). In addition, young patients were less likely to have heart failure on admission (10.5% vs 24.1%, P<0.001). No statistical significance was observed between the two groups in terms of in hospital morbidity except for congestive heart failure which was more predominant in older patients (6.1% vs 13.8%, P<0.001). The rate of coronary angiogram was significantly higher in younger patients (35.9% vs 32.2%, P=0.003). However, there was no statistical significance in PCI between the two groups (17.1% vs 14.2%, P=0.247). In ACS younger group in-hospital mortality rate (1.9% vs 4.8%, P<0.001), 30 days (4.4% vs 8.5%, P<0.001) and 1-year (5.7% vs 13.2%, P<0.001) were significantly lower than patients with ACS >40 years of age.

## Conclusions

ACS in young patients presents distinct risk factors and clinical characteristics. There is a need for prevention programs to control smoking epidemic by targeting young adults in the population. Our study document for the first time long-term outcome among young patients with ACS in the Middle East.

